# *O*-Linked Glycans of *Candida albicans* Interact with Specific GPCRs in the Coronary Endothelium and Inhibit the Cardiac Response to Agonists

**DOI:** 10.3390/jof9020141

**Published:** 2023-01-20

**Authors:** Alejandro Ocaña-Ortega, Gabriela Pérez-Flores, David Torres-Tirado, Luis A. Pérez-García

**Affiliations:** Facultad de Estudios Profesionales Zona Huasteca, Universidad Autónoma de San Luis Potosí, Romualdo del Campo 501, Fracc. Rafael Curiel, Ciudad Valles 79060, San Luis Potosí, Mexico

**Keywords:** *Candida albicans*, host-pathogen interaction, glycosylation, heart, endothelium, Langendorff model, GPCR, inotropic effect, vascular effect

## Abstract

*Candida albicans* is an opportunistic fungal pathogen that may cause invasive infections in immunocompromised patients, disseminating through the bloodstream to other organs. In the heart, the initial step prior to invasion is the adhesion of the fungus to endothelial cells. Being the fungal cell wall’s outermost structure and the first to come in contact with host cells, it greatly modulates the interplay that later will derive in the colonization of the host tissue. In this work, we studied the functional contribution of *N*-linked and *O*-linked mannans of the cell wall of *C. albicans* to the interaction with the coronary endothelium. An isolated rat heart model was used to assess cardiac parameters related to vascular and inotropic effects in response to phenylephrine (Phe), acetylcholine (aCh) and angiotensin II (Ang II) when treatments consisting of: (1) live and heat-killed (HK) *C. albicans* wild-type yeasts; (2) live *C. albicans pmr1*Δ yeasts (displaying shorter *N*-linked and *O*-linked mannans); (3) live *C. albicans* without *N*-linked and *O*-linked mannans; and (4) isolated *N*-linked and *O*-linked mannans were administered to the heart. Our results showed that *C. albicans* WT alters heart coronary perfusion pressure (vascular effect) and left ventricular pressure (inotropic effect) parameters in response to Phe and Ang II but not aCh, and these effects can be reversed by mannose. Similar results were observed when isolated cell walls, live *C. albicans* without *N*-linked mannans or isolated *O*-linked mannans were perfused into the heart. In contrast, *C. albicans* HK, *C. albicans pmr1*Δ, *C. albicans* without *O*-linked mannans or isolated *N*-linked mannans were not able to alter the CPP and LVP in response to the same agonists. Taken together, our data suggest that *C. albicans* interaction occurs with specific receptors on coronary endothelium and that *O*-linked mannan contributes to a greater extent to this interaction. Further studies are necessary to elucidate why specific receptors preferentially interact with this fungal cell wall structure.

## 1. Introduction

In recent years, the forms of infection of the genus *Candida* have been studied, since despite being a common microorganism within the native microbiota of humans, it can become an opportunistic pathogen in patients with chronic diseases who have received an organ transplant or undergone surgery [1,2]. It has also been shown that certain sectors of the population (older adults and newborns) are more prone to these fungal infections; this is because elderly people have a weakened immune system, and something similar happens in newborns, who still have a developing immune system [3,4]. These infections caused by *Candida* range from superficial mucosal to systemic mycosis, the latter with a mortality rate higher than 70% involving reaching the bloodstream and thus disseminating to different parts of the body such as bones, lungs, eyes, brain, kidneys, liver and heart, the latter being the most affected organ due to its direct connection with the bloodstream [5,6,7,8,9,10]. Invasive candidiasis is a major cause of morbidity and mortality in the healthcare environment, where risk factors such as the use of intravenous medical devices, immunocompromising conditions or prolonged therapies with broad-spectrum antibacterial drugs make patients vulnerable to *Candida* [5,11,12]. Once alterations in the mucosal microbiota and/or a weakening of the host immune system occur, *Candida* shifts from being a commensal to becoming an opportunistic, undergoing a morphological switch from yeast to hypha, thus enabling tissue invasion. Therefore, under such conditions, *Candida* might reach the bloodstream from mucosal surfaces [5]. Moreover, *Candida* has the ability to form biofilms on intravenous catheters and implantable medical devices in direct contact with the cardiovascular system, such as pacemakers, allowing the fungus to easily disseminate from these contaminated devices to the bloodstream, disseminating and potentially invading the rest of the body [5,13]. A possible complication of *Candida* colonizing the heart is the development of fungal endocarditis (FE), which remains the most severe form of infective endocarditis with a high mortality rate of about 50%. FE is usually diagnosed postmortem, since it is very difficult to identify the source, establish a diagnosis and carry out treatment [8,9,10,14,15]. The most frequently observed etiological fungi in FE are *Candida* and *Aspergillus* species; these can be isolated from surgically removed emboli, resected valves or infected foreign bodies [16]. Currently, the gold standard for the diagnosis of invasive candidiasis is a positive blood culture followed by light microscopy observation, and if FE is suspected, an echocardiography is also recommended [5]. Nowadays, *C. albicans* infections continue to be the leading cause of healthcare-associated systemic fungal infections, with mortality rates comparable to those of bacterial septicemia [4,17]. For all these reasons, it is of utmost importance to continue the investigation of the pathophysiological characteristics of *C. albicans* infection and to widen our knowledge of the interplay of this fungus with the heart, and the negative effects this interaction entails.

In the heart, initial and effective adhesion to the endothelial lining is required for posterior invasion of the cardiac tissue. It has been demonstrated that yeasts adhere significantly more to the endothelium under physiological flow conditions than hyphae and pseudohyphae [18]. The fungal cell wall is the first point of contact with host cells, apart from secreted proteins, and then follows its very important role in triggering the protective immune response and shaping the pathogen-host interplay that can lead to invasion or elimination. The *C. albicans* cell wall is mainly organized in two layers: an inner one close to the plasma membrane, mainly formed by chitin and β-(1,3) and β-(1,6) glucan, and an external layer formed by covalently attached proteins that are highly decorated by *O*-linked and *N*-linked mannans, consequently called mannoproteins [19]. Regarding adhesion, it is known that genes of the *C. albicans ALS* (agglutinin-like sequence) family encode cell wall GPI-anchored glycoproteins Als1–9, involved in the adhesion of the microorganism to the host cells as well as to other fungal or bacterial cells [20]. For example, it has been shown that the *C. albicans ALA1* gene product can bind to fibronectin, laminin and type IV collagen, all components either of the basal membrane or the extracellular matrix [21,22]. Nevertheless, although the Als proteins are thought to behave as lectins recognizing carbohydrate ligands, the data are insufficient to confirm such a statement [20]. Therefore, the mechanisms underpinning these interactions have not been entirely unveiled.

It has been reported that mannose polymers can bind to flow-sensitive lectinic ligands from the coronary endothelial luminal membrane, translating the coronary flow into cardiac responses [23]. G protein-coupled receptors (GPCRs), such as angiotensin II, α1-adrenergic and bradykinin receptors, among others, are reported to mediate coronary flow and agonist-induced responses via lectin-oligosaccharide interactions [24,25,26]. Thus, considering that (1) *C. albicans* has proven to bind to endothelium under flow conditions, and that in doing so it can cause conduction disturbances, and that (2) the lectinic nature of *C. albicans* cell wall may act as a natural source of mannose polymers, we investigated the effect of *C. albicans* yeast and its mannosylated cell wall structures on the function of flow-sensitive agonist-induced receptors in isolated perfused hearts. Our results showed that *C. albicans* interacts with the GPCRs angiotensin II and α1-adrenergic found in the coronary endothelium and block their function in response to their agonists in a mannose-dependent way. By perfusing isolated fungal cell walls and inactivated yeast cells, we found that the viability or integrity of the yeast cell is dispensable to achieve the cardiac effects produced by *C. albicans*. Furthermore, we found that this interaction depends only on the proper cell wall glycosylation, specifically of *O*-linked mannans, as live fungal cells from which these mannans have been chemically removed were unable to display the vascular and inotropic agonist-mediated effects that yeasts with intact *O*-linked mannans or isolated *O*-linked mannans elicited in the heart.

## 2. Materials and Methods

### 2.1. Strains and Culture Methods

The strains used in this study were *C. albicans pmr1*Δ (altered in the *O*-linked and *N*-linked mannosylation pathways) [27] and *C. albicans* NGY-152, which behaves like the wild-type strain [28]. Both strains were preserved and propagated at 28 °C in dextrose-Sabouraud medium (1% (*w/v*) yeast extract, 2% (*w/v*) mycological peptone, 2% (*w/v*) anhydrous glucose). For solid medium plates, 2% bacteriological agar was added. To prepare *C. albicans* yeast stock solutions, 500 µL were inoculated from an overnight pre-culture in 100 mL of fresh dextrose-Sabouraud medium and then incubated at 28 °C with constant shaking at 200 rpm (Shaking Incubator NB-T205, N-BIOTEK, INC., Bucheon-si, Republic of Korea 14502) until the exponential phase was reached (approximately 5 h). Cells were harvested by centrifugation at 2000× *g* (Sorvall legend XTR Thermo Scientific Centrifuge, Thermo Scientific, Osterode am Harz, Germany) and washed twice with sterile phosphate buffer saline (PBS, pH 7.4). Cell concentration was determined by cell counting in a Neubauer chamber and then diluted to a stock concentration of 1 × 10^8^ cells/mL in PBS (pH 7.4). For heat inactivation (HK), the yeasts were incubated in a thermoblock (Model FE400, Felisa, Jalisco, Mexico) at 65 °C for 1 h, during which time the cells retained wall and membrane integrity. The viability of the cells was confirmed by growth on dextrose-Sabouraud agar plates at 28 °C for 72 h. To corroborate membrane integrity, possible nucleic acid release was assessed by measuring the absorbance at 260 nm of the supernatant in a spectrophotometer (GenovaNano, JanWay Company, Cogan Station, PA, USA); values of A_260_ ≤ 0.020 were considered adequate. For heart experiments, yeast were diluted in Krebs–Henseleit solution (K-H, 127 mM NaCl, 6 mM KCl, 1.8 mM CaCl_2_, 1.2 mM NaH_2_PO_4_, 1.2 mM MgSO_4_, 25 mM NaHCO_3_, 5 mM dextrose, 2 mM pyruvate, pH 7.4) to a final concentration of 3.7 × 10^3^ cells/mL.

### 2.2. Fluorochrome Labeling

For chitin labeling, exponential phase cells were stained with WGA-FITC, 100 µg/mL (Sigma, Merck KGaA, Darmstadt, Germany). For mannan labeling, exponential phase cells were stained with 100 µg/mL ConA-FITC (Sigma, Merck KGaA, Darmstadt, Germany). Both were incubated at room temperature with gentle agitation and protected from light. After 30 min, the cells were washed twice with PBS and resuspended in 500 µL of PBS (Mora-Montes et al., 2011; Zhang et al., 2016). In parallel, heat-inactivated cells were stained with the same lectins coupled to fluorochromes. Preparations of 7 µL were observed with fluorescence microscopy using a Nikon^TM^ Eclipse Ni-U (Nikon Instruments Inc., Tokyo, Japan) fluorescence microscope. Image acquisition was performed with a Nikon^TM^ D3300 (Nikon Instruments Inc., Tokyo, Japan) camera. From the images taken of cells stained with WGA-FITC and ConA-FITC, green pixel counting was performed with Adobe Photoshop^TM^ 2022 software for 20 images in each condition and for each lectin used. The amount of fluorescence was determined in arbitrary units with the following formula: [(total green pixels − background green pixels) × 100]/total pixels [29].

### 2.3. Cell Wall Extraction

The cells were washed by centrifugation three times with cold deionized water at 2000× *g* for 5 min. The supernatant was discarded, and the cell pellet was resuspended in 500 µL of deionized water and stored at −20 °C in 1.5 mL microtubes. To each microtube, 0.4 g of glass beads were added, and then the cells were broken by vortexing for 1.5 h. The supernatant was recovered from the homogenate suspension. The supernatant was recovered in a new tube and washed by centrifugation at 2000× *g* for 5 min. The pellet was resuspended in 5 mL of 1M NaCl and washed by centrifugation 5 times with 1M NaCl. The pellet was finally resuspended in 5 mL of deionized water and aliquoted in 1 mL fractions into pre-weighed microtubes. All tubes were centrifuged at 13,000× *g* for 5 min, the supernatant was discarded, and the precipitate was resuspended in 1 mL of SDS/β-mercaptoethanol 0.3 M/EDTA 1M, pH 7.5/ Tris 50 mM. It was heated in a thermoblock at 100 °C for 10 min. It was centrifuged at 13,000× *g* for 5 min, washed with 1 mL of deionized water, and the supernatant was removed. The pellets were then frozen and lyophilized. The dry weight of the cell wall was determined, taking as a reference the initial weights of the tubes [30]. For interaction, the walls were resuspended in K-H solution at a final concentration of 1 mg/mL and just before perfusion, they were sonicated (Fisherbrand™ Model 120 Sonic dismembrator, Fisher Scientific, Pittsburgh, PA, USA) employing four cycles of 1-min 120 W and 20 kHz with 100% of amplitude to break wall aggregates.

### 2.4. Extraction and Purification of Glycans

To obtain cell wall glycans, yeast from an exponential phase culture of *C. albicans* WT was harvested by centrifugation and washed three times with sterile deionized water. To remove *N*-linked glycans, the pellet was resuspended in 25 mL of a solution of 120 mM NaOAc and 500,000 U/mL endoglycosidase H (New England Biolabs) and incubated overnight at 37 °C without shaking. After this time, the cells were harvested by centrifugation at 2000× *g* for 5 min, the supernatant containing the *N*-linked glycans was collected, and the pH was adjusted to 7.0 with 100 mM NaOH. The cell package was kept frozen at −20 °C for interaction studies [31]. *O*-linked glycans were obtained by β-elimination by resuspending the cell pellet in 10 mL of 100 mM NaOH and were incubated overnight at room temperature (25 °C) with shaking at 30 rpm. The samples were centrifuged at 2000× *g*, the supernatant was collected, and the pH was adjusted to 7.0 with 100 mM HCl. The cell package was kept frozen at −20 °C [32]. The glycans recovered in the supernatants were purified separately by adding an equal volume of Fehling’s reagent and subjected to heating to precipitate mannan, creating a mannan-copper complex. The supernatant was decanted, and 8 mL of 3M HCl was added. The solution was poured into 100 mL of methanol-acetic acid 8:1 (*v/v*) until the liquid turned green and allowed to stand at room temperature for several hours until sedimentation of the precipitate. The liquid was filtered, and the precipitate was recovered and dissolved again with methanol-acetic acid 8:1 (*v/v*). The dissolution and precipitation procedures were repeated until the methanol-acetic acid mixture stopped turning green. Subsequently, the mixture was washed with methanol and ethyl ether and allowed to dry at room temperature. Finally, the mannan sediment was separated by filtration and dissolved in distilled water [33]. The purified mannans were determined for protein content by the Bradford method, where a protein concentration of less than 1 mg/mL was considered acceptable for use. The mannans were placed in pre-weighed tubes to be lyophilized, then their dry weight was determined, and they were resuspended in K-H solution to a final concentration equivalent to 10 mM mannose. Before perfusion, the glycans were sonicated to eliminate possible aggregates, as already described for the cell walls.

### 2.5. Isolated Heart Model

The Langendorff’s isolated heart model is widely accepted to perform physiological tests in the heart, since it allows to study the organ in absence of other body systems, exocrine control and neurohumoral factors that may bewilder physiological measurements in the intact animal [34,35]. In this model, the heart is perfused by the aorta to produce an aortic outflow that closes the aortic valve and infuses the solution directly into the coronary tree [35]. For Langendorff’s model, we used male Wistar rats (300 to 400 g) provided by the Bioscience Center, Universidad Autónoma de San Luis Potosí (UASLP). All animals were kept in cages at a controlled temperature (25 °C, aprox.), light/dark cycles (12 h), and with food and water *ad libitum*. The experiments were conducted in accordance with the Mexican guidelines for the production, care and use of laboratory animals [36]. Animals were anesthetized with sodium pentobarbital (50 mg/kg) and sodium heparin (500 IU) intraperitoneally. The rat underwent a thoracotomy to expose and remove the heart, which was immediately placed in a cold K-H solution. Once the heart was isolated, the ascending aorta was located and attached to a non-recirculating retrograde perfusion system (Langendorff´s method) using a cannula. The heart rate was maintained at 4.5 Hz by applying electrical square pulses (S44 Stimulator, Grass Instrument Co., West Warwick, RI, USA) using a pair of electrodes, and then a constant flow of 8 mL/min was established with physiological K-H solution. The K-H solution was continuously bubbled with a mixture of 95% O_2_ and 5% CO_2_ at pH 7.4 and a temperature of 37 °C. After the above conditions were established, a stabilization period of 10 min was waited before measurement of any parameter or application of the treatment.

### 2.6. Coronary Perfusion Measurement

Once the heart was placed in the system, a pressure transducer (TSD104A Model MP150, BIOPAC Systems, Inc., Goleta, CA, USA) was connected to a side branch of the perfusion cannula, which allowed measurement of the coronary perfusion pressure (CPP) at a flow rate of 8 mL/min and thus determined the coronary vascular resistance [37].

### 2.7. Measurement of Left Ventricular Contraction

The inotropic effects (force of contraction) were determined from measurements of changes in left ventricular pressure (LVP). For this purpose, a latex balloon attached to a pressure transducer was placed in the left ventricle to measure LVP, after ventricular contraction, pressure developed, and its amplitude was continuously recorded. All changes in the amplitude of LVP developed were taken as a qualitative indicator of the inotropic response [37].

### 2.8. Evaluation of Vascular and Inotropic Responses Induced by Intracoronary Administration of Acetylcholine, Phenylephrine and Angiotensin II

**Figure 1:**
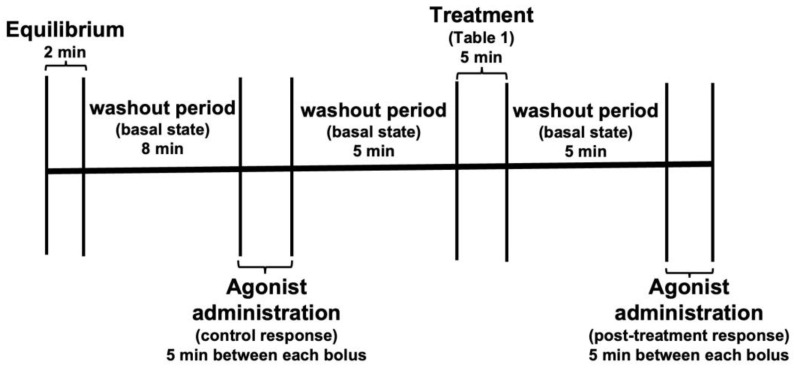
A representative scheme of the experimental flow used to assess the effect of the treatments on the cardiac response to agonists. Once the heart was attached to a non-recirculating retrograde perfusion system, a 10 min-period was given for stabilization (basal state) prior to the administration of Ang II, Phe and aCh to register the effects of agonists on the heart (control response over the basal state of the heart). Treatment was administered for 5 min after a second stabilization period and then extensively washed out to eliminate the non-adhered cells/structures. Then, a second administration of the agonists was performed to determine the effect of the treatment on the cardiac response (response post-treatment). For the initial experiment with *C. albicans* WT, 10 mM mannose was administered to the heart, followed by a third administration of the agonists to record the effect exerted by this sugar on reverting the treatment effect on cardiac responses (not shown).

**Figure 2:**
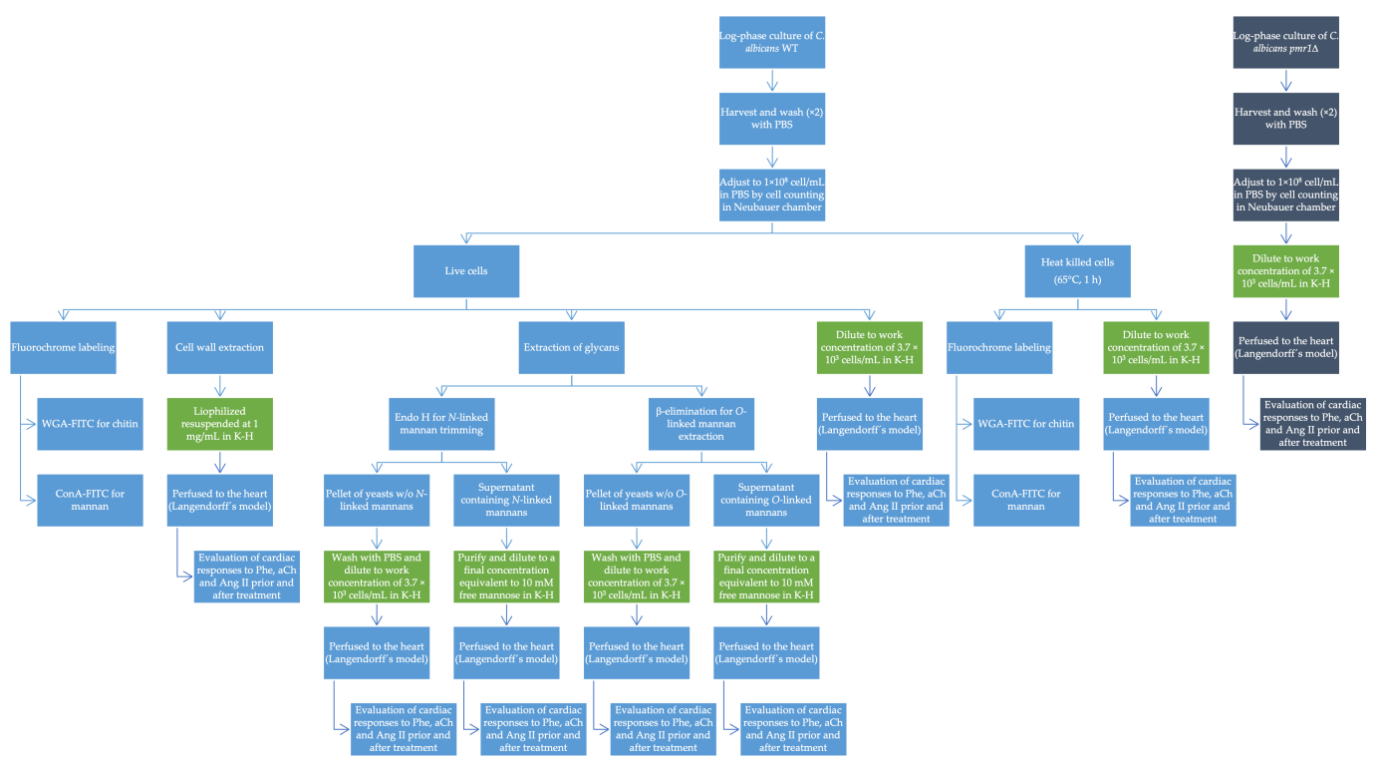
Diagram showing the general flow of the experimental procedures used in this study. Experiments performed with *C. albicans* WT are shown in light blue boxes. Experiments performed with *C. albicans pmr1*Δ are shown in dark blue boxes. The work suspensions/solutions for the treatments described in Table 1 are shown in green boxes.

To study the effects of *C. albicans* and its different subcellular structures exerting on the heart, the latter was given a bolus of 30 μL of phenylephrine (Phe, 1 × 10^−6^ M) and the changes in LVP and CPP variables were recorded, after 5 min 30 μL, of acetylcholine (aCh, 1 × 10^−6^ M) was administered, and again the change in variables was recorded, subsequently 30 μL of angiotensin II (Ang II, 1 × 10^−6^ M) was administered and the changes in variables were recorded. These initial responses were taken as the control response and normalized as a percent over the basal state of the heart before any treatment. After recording the control responses, the heart was administered the corresponding treatment (Table 1) for 5 min at a constant flow rate of 8 mL/min, and then the drug administration was repeated as described in the previous paragraph. LVP and CPP variables were recorded to compare responses in the presence and absence of each treatment (Figure 1). After this, only the interaction with *C. albicans* WT, a 10 mM mannose wash, and a third administration of the drugs and the corresponding recording of LVP and CPP variables were performed. For each heart used, two responses were obtained for each drug applied: One in the normal condition of the heart (response 1) and the other after each treatment (response 2). Response 1 was calculated as the 100% response, and response 2 was calculated as a percentage of response 1. In the case of *C. albicans* WT, a third response was obtained for each drug and was calculated as a percentage of response 1. Figure 2 depicts a general diagram of the experimental procedures used in this study.

### 2.9. Statistical Analysis

Statistical analysis and graphs were performed with GraphPad Prism 8 software for Windows. The data represented in the LVP and CPP graphs correspond to the percentage of the response for each drug, before and after each treatment. Results are expressed as the mean ± standard error and analyzed by a two-way ANOVA. Fluorescence data were analyzed by an unpaired t-test. In all cases, a *p*-value less than 0.05 was considered statistically significant.

## 3. Results

### 3.1. C. albicans Inhibits the Cardiac Response to Phe and Ang II but Not to aCh in a Mannose-Dependent Way

**Figure 3:**
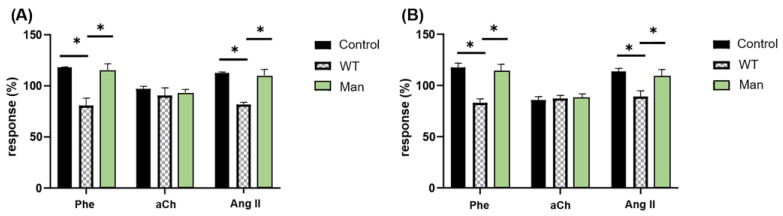
*C. albicans* interacts with coronary endothelium and inhibits the effects of agonist-induced GPCRs, and this inhibition is reversed by mannose. (**A**) Vascular and (**B**) inotropic effects of Phe, aCh and Ang II agonists were determined at the beginning of the experiment (Control), as well as after interaction with *C. albicans* WT yeasts (WT) and after washout with mannose (Man). Bars (mean ± SD) show the result of at least three independent experiments (* *p* < 0.05).

As described, a simple and accurate way to assess the function of a G protein-coupled receptor (GPCR) is the measurement of the response induced by its activation with a specific agonist. To observe the effect of *C. albicans* WT on the activation of GPCRs by agonists, it was decided to measure the responses to Phe, aCh and Ang II by administering them at a submaximal dose (95% max) for receptor activation [37]. The effects were determined by measuring the response in two different variables: the inotropic effect (measuring LVP) and the coronary vascular response (measuring CPP). As expected, both Phe and Ang II had a positive effect on the control response of both variables (LVP and CPP), evidencing their vasoconstrictor activity; unlike aCh, which showed a negative effect on the control response of LVP and CPP, consistent with its activity as a vasodilator. After perfusion of *C. albicans* WT yeast to the heart, the magnitudes of these responses decreased for Ang II and Phe compared to the control responses, while for aCh they remained without significant change. This *C. albicans* WT blockage on LVP and CPP in response to Phe and Ang II agonists was restored after mannose infusion (Figure 3). These results indicate that *C. albicans* WT is capable of interacting with the coronary endothelium and altering the parameters of CPP (Figure 3A) and LVP (Figure 3B), in response to agonists such as Phe and Ang II but not aCh. In addition, it is evident that this interaction is reversible when washing with mannose is carried out, so we can assume that this interaction is mediated by mannose-rich structures.

### 3.2. The Interaction of C. albicans with Specific GPCRs Is Due to the Cell Wall Structure and Not to Secreted Components

**Figure 4:**
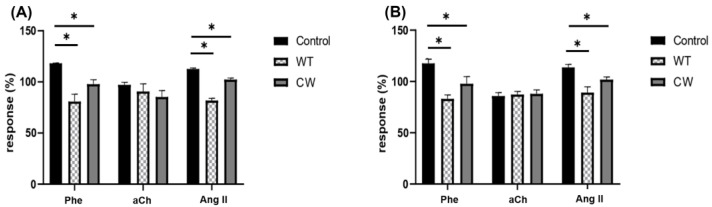
Modification of CPP and LVP in response to agonists when *C. albicans*’ cell wall interacts with coronary endothelium. (**A**) Heart response, expressed as percentage of response of CPP and (**B**) LVP, when Phe, aCh and Ang II are administered in the absence (control) and in the presence of *C. albicans* WT yeasts (WT) or purified *C. albicans* WT cell walls (CW). Bars (mean ± SD) show the result of at least three independent experiments (* *p* < 0.05).

Once we observed that *C. albicans* interacted with specific receptors of the coronary endothelium, modifying parameters such as CPP and LVP in response to drugs such as Phe and Ang II, we were interested in knowing whether this interaction was due entirely to the fungal cell wall, which is the most external point of contact of the fungus with host cells, or whether it was due to some secreted metabolite or even to a spatial impediment where the yeasts were attached to another structure of the endothelium that, due to its proximity to the receptors studied because of the size of the yeast, prevented the interaction of the drug with its receptors. For this purpose, exponential phase *C. albicans* yeast cell walls were extracted and extensively washed with an ionic solution to eliminate any protein in transit other than those covalently attached to the cell wall, and then perfused to the heart at a concentration of 1 mg/mL, and CPP and LVP variables were measured in response to the agonists. A heart treated with live *C. albicans* yeast was used as a control for comparison of the response of the isolated walls (Figure 4).

The results showed that the cell wall of *C. albicans* alone, in the absence of possible secreted metabolites and without having the three-dimensional conformation of yeast, is able to modify CPP (Figure 4A) and LVP (Figure 4B) in response to agonists such as Phe and Ang II but not aCh at levels comparable (*p* > 0.05) to those obtained when the whole yeast interacts, indicating that *C. albicans* interaction is specifically due to the cell wall structure and that the inhibition of cardiac responses to Phe and Ang II is due to the presence of this fungal structure and not the spatial conformation of the yeast, which could block the receptors by adhesion to structures proximal to them.

### 3.3. Proper C. albicans Glycosylation Is Required for Its Interaction with Specific GPCRs of the Coronary Endothelium

Once it was observed that the cell wall was able to induce the same effect as whole yeast, we set out to confirm that the mannosylated structures (*N*-linked mannans and *O*-linked mannans) present in the wall were important in facilitating the interaction of the fungus with the coronary endothelium, since the cell walls when isolated could interact with the heart both through the inner layer (rich in chitin and glucans) and the outer layer (rich in mannose). We used two approaches to confirm this hypothesis.

**Figure 5:**
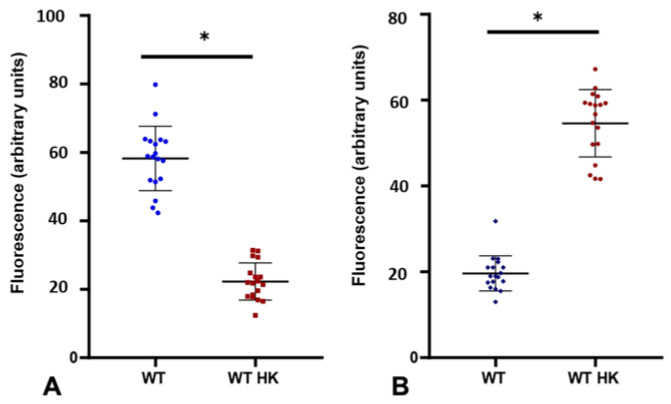
Exposure of mannan and chitin in *C. albicans* yeasts. From the images acquired from ConA-FITC (**A**) and WGA-FITC (**B**) staining experiments. The exposure of (**A**) mannan in live (blue dots) and heat-innactivated (HK, red squares) wild type (WT) cells, as well as the (**B**) chitin in live (purple diamonds) and heat-inactivated (HK, red dots) wild type (WT) cells, was calculated based on the fluorescence that the cells exhibit. The graph (mean ± SD) shows the fluorescence results of 20 different images for each treatment (* *p* < 0.0001).

One of them was using heat-inactivated *C. albicans* cells (*C. albicans* HK), which are yeasts of the WT strain that, when heated at 65 °C, alter the organization of their cell wall, exposing the internal components of the cell wall, i.e., chitin and β-glucans, while internalizing the mannans that are usually found in the outer surface layer. To corroborate this effect of temperature, WGA-FITC labeling was used for chitin, since the wheat germ lectin WGA (from *Triticum vulgaris*) has been described to bind mainly to *N*-acetylglucosamine (GlcNAc), the main component of chitin. On the other hand, ConA-FITC was used to label mannans, as concanavalin A (ConA) binds predominantly to terminal α-mannose residues [38]. Both of these tags were applied to live and heat-inactivated (HK) *C. albicans* WT cells. The level of exposure to these components, both chitin and mannose, was quantified indirectly by performing an analysis of the captured fluorescence microscopy images. The green pixels corresponding to fluorescence were quantified using image analysis software and plotted for both treatments (Figure 5).

**Figure 6:**
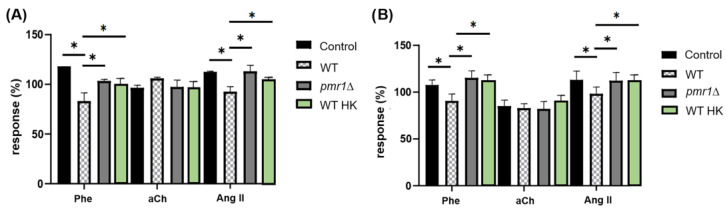
Modification of CPP and LVP in response to agonists when *C. albicans* HK and *C. albicans pmr1*Δ interact with the coronary endothelium. Heart response, expressed as percentages of (**A**) CPP and (**B**) LVP, when Phe, aCh and Ang II are administered in the absence (Control) and in the presence of *C. albicans* WT (WT), heat-inactivated *C. albicans* (WT HK) or *C. albicans pmr1*Δ (*pmr1*Δ) yeast. Bars (mean ± SD) show the result of at least three independent experiments (* *p* < 0.05).

Pixel analysis for mannose exposure (Figure 5A) corroborates that when cells are heat-inactivated, external components such as mannan are less exposed and thus less accessible to interaction with the ConA-FITC molecule and hence with coronary endothelium. Internal components such as chitin that are less exposed in living cells, upon inactivation by heat, remain on the surface and are therefore more accessible to interaction with the WGA-FITC molecule, in contrast to mannan and ConA-FITC. Once it was proven that heat-inactivated cells reverse the distribution of their cell wall components, we proceeded to perform experiments perfusing *C. albicans* HK yeasts to determine the effect they had on CPP and LVP (Figure 6) in response to agonists.

The second approach we used was to address the interaction of coronary endothelium with *C. albicans pmr1*Δ cells. This strain has a disruption in the *PMR1* gene encoding a calcium (Ca^2+^) and manganese (Mn^2+^) ion pump, both cofactors of the mannosyltransferases responsible for glycosylation pathways. In the absence of this pump, there is a cofactor deficiency, and the mannosyltransferases cannot add mannose residues, so their *N*-linked mannans lack the outer chain and *O*-linked glycans are truncated after the first mannose residue [27]. In both cases, with *C. albicans* HK and *C. albicans pmr1*Δ, *C. albicans* WT yeasts were used as controls to compare the responses.

We observed that neither *C. albicans* HK nor *C. albicans pmr1*Δ were able to alter CPP (Figure 6A) and LVP (Figure 6B) parameters in response to agonists, as they do not show a significant difference when compared to the control response for Phe and Ang II, both agonists displaying a clear effect when *C. albicans* WT and isolated cell walls are perfused to the heart. These results indicate that *C. albicans* cell wall mannoproteins are indispensable for interaction with coronary endothelial GPCRs, since *C. albicans* HK cells that do not expose mannoproteins on their surface are unable to inhibit the agonist responses that cells with a properly organized cell wall accomplish. Likewise, proper mannosylation of *C. albicans* is necessary to achieve interaction with the coronary endothelium since *C. albicans pmr1*Δ yeast with truncated mannans do not alter the parameters studied in response to agonists as observed with the wild-type strain.

### 3.4. O-Linked Mannans from C. albicans Cell Wall Inhibit the Agonist-Mediated Response of GPCRs in the Coronary Endothelium

**Figure 7:**
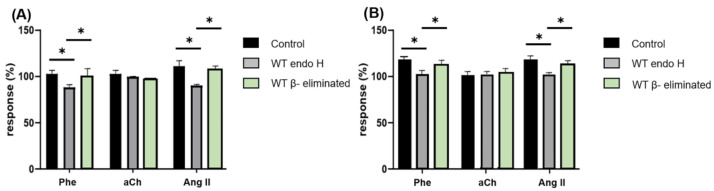
Modification of CPP and LVP in response to agonists when interacting *C. albicans* without *N*-linked mannan and *C. albicans* without *O*-linked mannan with coronary endothelium. Heart response, expressed as percentages of (**A**) CPP and (**B**) LVP, when Phe, aCh and Ang II are administered in the absence (Control) and in the presence of *C. albicans* without *N*-linked mannans (WT Endo H) or *C. albicans* without *O*-linked mannans (WT β-eliminated). Bars (mean ± SD) show the result of at least three independent experiments (* *p* < 0.05).

Once we verified that the external mannosylated structures of the cell wall were responsible for the observed effects on the response to agonists, we proceeded to isolate, separately, *N*-linked mannans and *O*-linked mannans from the cell wall of live *C. albicans* yeasts to evaluate the effect of the yeast without *N*-linked mannan, yeast without *O*-linked mannan, as well as *N*-linked and *O*-linked mannans isolated separately, on the heart. *N*-linked mannans were extracted by an enzymatic process catalyzed by endoglycosidase H (Endo H), which hydrolyzes the glycosidic bond between the two GlcNAc residues of the *N*-linked mannan core, leaving only one GlcNAc residue attached to the protein sequon [31]. For the extraction of *O*-linked mannans, a β-elimination in an alkaline medium was performed, which, by a chemical process, broke the ester bond between the first sugar residue and the amino acid [32]. These extractions render the complete viable yeast but without the *N*-linked and *O*-linked mannans, respectively, which were perfused to the heart to evaluate the effect of cells lacking each type of mannans (Figure 7).

**Figure 8:**
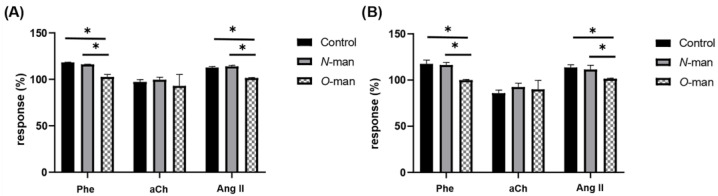
Modification of CPP and LVP in response to agonists when interacting isolated *C. albicans* mannans with coronary endothelium. Heart response, expressed as percentages of (**A**) CPP and (**B**) LVP, when Phe, aCh and Ang II are administered in the absence (Control) and in the presence of *C. albicans N*-linked mannans (*N*-man) or *C. albicans O*-linked mannans (*O*-man). Bars (mean ± SD) show the result of at least three independent experiments (* *p* < 0.05).

Separately extracted and purified *N*-linked and *O*-linked mannans were determined for purity by protein quantification (>1 mg protein/mL), thus confirming that the mannan had been correctly separated from the cell wall proteins [31]. The purified mannans were used in independent experiments to evaluate the effect they had on CPP and LVP modification in response to agonists. For this purpose, the effect of Phe, Ang II and aCh on these two parameters (Figure 8) was evaluated before and after interaction with *N*-linked or *O*-linked mannans.

**Figure 9:**
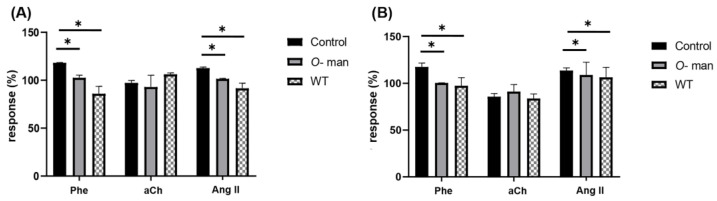
Modification of CPP and LVP in response to agonists when interacting isolated *C. albicans* mannans with coronary endothelium. Heart response, expressed as percentages of (**A**) CPP and (**B**) LVP, when Phe, aCh and Ang II are administered in the absence (Control) and in the presence of *C. albicans N*-linked mannans (*N*-man) or *C. albicans O*-linked mannans (*O*-man). Bars (mean ± SD) show the result of at least three independent experiments (* *p* < 0.05).

As it can be observed in Figure 7, *C. albicans* without *N*-linked mannan still affects the CPP and LVP in response to Phe and Ang II, a similar effect to that observed with *C. albicans* WT, conversely yeasts lacking *O*-linked mannans do not alter the response of any of the three agonists. In accordance with these observations, isolated *N*-linked mannans do not exhibit any effect on the responses to agonists, unlike isolated *O*-linked mannans that were able to achieve by themselves the same effect as *C. albicans* WT (Figure 9). These results indicate that *O*-linked mannans are mediating the interaction with the coronary endothelium and inhibiting the response of Phe and Ang II receptors.

## 4. Discussion

*C. albicans* is the most frequent etiological agent causing candidemia in immunocompromised patients, reaching mortality rates ranging from 50% to 70% [5,39]. It has the ability to form biofilms on catheters and other implantable medical devices, thus acting as a reservoir for fungal cells that can be later disseminated [13]. When such biofilms occur on peripheral or central vascular catheters and pacemakers, *Candida* eventually comes into contact with the heart [40,41]. Efficient adherence and further invasion of *C. albicans* in epithelial or endothelial cells is a very important trait for the virulence of this fungus, along with the morphological switch from yeast to hypha and secreted proteases and phospholipases, all contributing to tissue damage and organ invasion [5,42].

Here, we show that *C. albicans* interacts with the coronary flow-dependent GPCRs α1-adrenergic and angiotensin II receptors, inhibiting their agonist-mediated effects on cardiac function, and such effects can be entirely annulled by perfusion of mannose (Figure 3). These observations are similar to those reported for *Candida glabrata*, where the agonist-mediated effects caused by the fungus were totally reversed by mannose and, to lesser extent, by galactose [37]. This suggests a mannose-mediated interaction, and considering that the endothelial membrane has numerous lectins and glycosylated proteins [23,24,25], it is possible that reciprocal lectinic-oligosaccharide interactions similar to those described for fungal immune sensing by C-type lectin receptors recognizing carbohydrate pathogen-associated molecular patterns might be involved in the alteration of the function of these GPCRs in the presence of *C. albicans* [43]. Interestingly, for the muscarinic receptor of aCh, with vasodilator effect, neither changes in CPP nor in LVP in the agonist-mediated response were altered due to the presence of *C. albicans*, in accordance with what has been observed for bradykinin, another vasodilator, with *C. glabrata* [37].

It has been mentioned above that the fungal cell wall is the first point of contact with host cells; nevertheless, fungal cells also possess secreted virulence factors aiming to inactivate host defensive traits and to degrade the host tissue, favoring invasion [21]. To explore the role of the fungal cell wall regarding the interaction with coronary endothelium, *C. albicans* cell walls were perfused to the heart, thus avoiding the possible effect due to secreted proteins. Results demonstrated that the cardiac effect provoked by *C. albicans* can also be induced by the cell wall itself (Figure 4), indicating that the putative ligand or ligands responsible for the interaction and consequent cardiac effect belonged to the fungal wall, more likely of a saccharidic nature. Moreover, interaction of the heart with heat-inactivated fungal cells, revealed that cell wall organization, namely mannoprotein exposure (Figure 5), instead of the three-dimensional structure of *C. albicans* yeasts, is relevant to observe the same effect on cardiac responses as that induced by *C. albicans* WT (Figure 6), reinforcing the hypothesis of a lectinic-oligosaccharide interaction [23,24]. *C. albicans pmr1*Δ is a null mutant displaying truncated *O*-linked glycans and *N*-linked glycans lacking the outer chain. The cardiac response after the perfusion of this strain allows us to confirm the important role of fungal mannosylation to enable the interaction of *C. albicans* with the coronary endothelium, since any significant change was recorded in the CPP or LVP in response to agonists after *C. albicans pmr1*Δ administration compared to the control response. In other words, fungal cells lacking proper mannosylation cannot interact with the coronary endothelium. Our data contrast with those previously reported by Bates et al. (2005), where authors mention that *C. albicans pmr1*Δ has attenuated virulence compared to the WT strain and that its cell wall is more permeable but that its adherence to buccal epithelial cells is not significantly affected [27]. On this discrepancy related to adhesion, it could be commented that in our work the possible adherence of *C. albicans pmr1*Δ would be given by Phe (α1-adrenergic) and Ang II receptors, which are not expressed in the buccal epithelium, suggesting that interaction and further adhesion of *Candida* cells occur differently depending on the host tissue and that different receptors and mechanisms might be involved, as it has been previously proposed [22].

Using different approaches, either perfusing yeast cells without each type of mannan structure (Figure 7) or the isolated *N*-linked and *O*-linked mannans (Figure 8), we determined that the latter were the structures accountable for the interaction with the coronary endothelium and the observed cardiac effects. Likewise, isolated *O*-linked glycans were able to reproduce similar effects to those exerted by *C. albicans* WT (Figure 9). This phenomenon is particularly relevant, since it suggests that prior to fungal direct contact with the heart, cardiac agonist-mediated responses can be triggered by soluble mannose oligosaccharides released from the fungal cell wall through natural shedding.

Our data support the idea that the mannosylated structures of the cell wall are indispensable components for the interaction of *C. albicans* with the coronary endothelium (Figure 6, Figure 7, Figure 8 and Figure 9). These results are in agreement with those described by Borong et al. (2020), where the authors stated that in pathogen-host interactions, glycoproteins are the main structures that promote adhesion and then cause infection in the host [44]. Considering what was reported by Bates et al. (2005), it could be thought that the adhesion of *C. albicans* to host tissues is mediated by different structures of both the pathogen and the host, depending on the anatomical site where the interaction occurs, which broadens the possibilities of interaction in different tissues, allowing a great diversity in the mechanisms of infection [27]. One of the first studies addressing the interaction of *C. albicans* with the coronary endothelium was described by Scheld et al. (1985), where they discuss the in vitro adherence of various microorganisms (including *C. albicans*) to fibronectin, which correlates with endocarditis in rabbits [45]. Nonetheless, the interaction of *Candida* does not always occur on damaged tissue as was explored in our work, where the hearts came from healthy rats, so it is unlikely that there were lesions in the coronary endothelium that would expose fibronectin. Klotz et al. (1992) investigated a peptide containing arginine, glycine and aspartic acid (RGD) in hematogenous *C. albicans* infections in rabbits. In that study, the authors mention that *C. albicans* has strong adhesion to numerous human proteins such as fibrinogen, laminin and fibronectin and iC3b, which may be related to the pathogenicity of this microorganism since all these proteins contain the RGD sequence, a ligand recognized by many mammalian integrins. The authors suggest that the amino acid sequence of RGD can be recognized by receptors in *C. albicans* [46]. Considering that our results show a selectivity of *C. albicans* to interact with Ang II and Phe receptors, we proceeded to explore the possibility that these receptors present the RGD sequence; however, neither of the two receptors whose function is affected by the presence of *C. albicans* possesses in its amino acid sequence the RGD peptide (data not shown). Similarly, the muscarinic receptor for aCh, whose function was not affected in our experiments, does not have RGD in its sequence either (data not shown). Since that these reports deal with the adhesion of *C. albicans* to proteins present in the extracellular matrix (ECM) or in the basement membrane of the endothelium, whose exposure occurs only in the glomerular vasculature (fenestrated vessels) or after damage to the endothelium [22,46], we could consider that this interaction mechanism is not the one used by the fungus to adhere to healthy coronary endothelium before starting a tissue invasion, meaning that the yeast interacts with the tissue by another mechanism and once it has caused damage with the other virulence factors, it maintains adhesion by binding to ECM proteins that have the RGD peptide in their sequence, thus perpetuating its adhesion and favoring the progression of the infection. The idea that there are several mechanisms allowing the interaction and subsequent adhesion of *C. albicans* with host tissues has already been explored by several authors. Based on studies in which fungal yeasts were co-incubated with buccal epithelial cells (BEC), it was described that there was a strain-specific lectin-oligosaccharide type interaction where *C. albicans* mannoproteins could bind to fucose-galactose and/or *N*-acetylglucosamine-galactose residues [47].

Considering that *O*-linked mannans were able to produce the same effect as whole yeast (Figure 9), we can suggest these structures and not *N*-linked mannans as the ones responsible for the interaction of *C. albicans* with GPCRs of the coronary endothelium. This observation is consistent with previous studies reporting that null mutants of *C. albicans* in the *pmt1*Δ and *pmt2*Δ genes, involved in the construction of *O*-linked glycans, presented cells with truncated *O*-linked mannans unable to adhere to both epithelial cells (BEC and vaginal epithelial cells) and matrigel (commercial basement membrane matrix-like product composed mainly of laminin, followed by collagen IV, heparan sulfate proteoglycans, entactin/nidogen and various growth factors) [48]. These results are in agreement with previously reported lectin-oligosaccharide (in the epithelium) and RGD-peptide binding (in matrigel) adhesion mechanisms [47], but are not entirely comparable with our observations once the receptors involved in the coronary interaction are absent in those cell lines and definitely are not found in Matrigel. Although the lectin-like behavior of Als proteins to recognize carbohydrate ligands is not entirely confirmed, it has also been suggested based on amino acid sequence analysis that a region rich in serine and threonine residues is a highly potential candidate to undergo *O*-linked manosylation [18], implying that these proteins and, more specifically, their glycosylation, could be responsible for the cardiac effects observed in our results.

## 5. Conclusions

This work has demonstrated the importance of *C. albicans* glycosylation in its interaction with the coronary endothelium, specifically affecting cardiac CPP and LVP parameters in response to vasoconstrictor drugs such as Phe and Ang II but not vasodilators such as aCh when yeasts are present in the cardiac vasculature.

It was demonstrated that fungal fractions, such as the cell wall or isolated *O*-linked mannans, are able to achieve the effect obtained by *C. albicans* WT when interacting with the endothelial membrane. Opposing this, it was observed that *C. albicans* HK (which presents alterations in the organization of its cell wall), *C. albicans pmr1*Δ (which has defects in its *N*-linked and *O*-linked glycosylation), and isolated *N*-linked mannans are not able to interact with the Phe and Ang II receptors of the coronary endothelium.

Taken together, our results allow us to conclude that cell wall *O*-linked mannans are the structures mediating the interaction of *C. albicans* with the coronary endothelium via Phe and AngII receptors (GPCRs for vasoconstrictor agonists), affecting the vascular and inotropic responses of the heart mediated by these agonists. These data do not rule out that *N*-linked mannans could mediate the interaction of the fungus with receptors other than those studied here, so it is important to expand the studies to elucidate the nature of this interaction and the reason why some receptors bind preferentially to one or another structure of the fungal cell wall.

## Figures and Tables

**Table 1:** Strains and structures of *C. albicans* used for interaction with the coronary endothelium.

Treatment	Description	Reference
*C. albicans* WT (NGY-152) (3.7 × 10^3^ cel/mL)	*ura3*Δ::imm434/*ura3*Δ::imm434, *RPS1*/*rps1*Δ::CIp10. It behaves like the wild-type strain	[28]
*C. albicans* WT HK (3.7 × 10^3^ cel/mL)	*ura3*Δ::imm434/*ura3*Δ::imm434, *RPS1*/*rps1*Δ::CIp10. It behaves like the wild-type strain and it was heat-inactivated	[28]
*C. albicans pmr1*Δ (3.7 × 10^3^ cel/mL)	*ura3*Δ::imm434/*ura3*Δ::imm434; *pmr1*Δ::*hisG*/*pmr1*Δ::*hisG*, *RPS1*/*rps1*Δ::CIp10. It has truncated both *N*-linked and *O*-linked mannans.	[27]
*C. albicans* without *N*-linked glycans	*C. albicans* WT (NGY-152) treated with endo H	[31]
*C. albicans* without *O*-linked glycans	*C. albicans* WT (NGY-152) β-eliminated	[32]
*N*-linked glycan (10 mM)	Glycans recovered and purified from *C. albicans* WT trimming with Endo H	[31,33]
*O*-linked glycan (10 mM)	Glycans recovered and purified from β-elimination of *C. albicans* WT	[32,33]
Cell wall from *C. albicans* WT (1 mg/mL)	Purified yeast cell walls of *C. albicans* WT (NGY-152)	[30]

## Data Availability

Not applicable.
